# Editorial: The Trigeminocardiac Reflex: Beyond the Diving Reflex

**DOI:** 10.3389/fnins.2017.00673

**Published:** 2017-12-01

**Authors:** Bernhard Schaller, Tumul Chowdhury, Thomas Rosemann

**Affiliations:** ^1^Department of Primary Care, University of Zurich, Zurich, Switzerland; ^2^Department of Anaesthesiology and Perioperative Medicine, University of Manitoba, Winnipeg, MB, Canada

**Keywords:** trigeminocardiac reflex, models, theoretical, skull base surgery, neuroanesthesiology, neuroanatomy

The trigeminocardiac reflex (TCR) is a well-described and also well-known brainstem reflex that is extensively researched and reported in clinical neurosciences during the last nearly 20 years (Schaller et al., 1999). During this time period, investigators have explored the physiological and/or pathological, but also the neurobiological nature of this unique reflex (Schaller, 2004; Filis et al., 2008; Schaller et al., 2008a, 2009b; Arasho et al., 2009; Meuwly et al., 2013; Lemaitre et al., 2015) as well as its consequences on various surgical outcomes (Gharabaghi et al., 2006; Schaller et al., 2007, 2008b). In addition, albeit few animal experiments on the TCR models also provided its relationship with cerebral hemodynamics and metabolism (Sandu et al., 2010; Lapi et al.; Buchholz et al.) and included the concept of oxygen conserving reflex into the TCR (Schaller et al., 2009a). Currently, we are in a new phase of the TCR research: We have to understand in which kind of diseases the TCR might also play a role and how we could utilize this information for developing future interventions and treatment modalities (see for example, Cornelius et al., 2010). It is therefore the time to reflect what we have achieved in the TCR research so far.

From the very beginning, the TCR was considered as the most powerful autonomous reflex in humans and mammals. For a substantial time-span, the principal knowledge of the TCR was especially and nearly exclusively related to a fundamental work written in 1999 that introduced this reflex into various neurosurgical procedures, especially of the skull base (Schaller et al., 1999). The scientific evidence of the reflex's validity/reliability was provided on a causal relationship basis, and the TCR arc was described based on the trigeminal and cardioinhibitory vagus nerves as the afferent and efferent pathways respectively (Schaller et al., 1999). This initial case series introduced, for the first time, an emergent TCR definition based on clinical, but also theoretical consideration. Thereafter, the in the following years published case reports could focus mainly on the differentiation between the peripheral and the central stimulation (Schaller et al., 2009b) providing strong evidence that the peripheral triggered TCR (via the spinal nucleus of the trigeminal nerve to the Kölliger-Fuse nucleus) is different from the TCR triggered by central stimulation (via the nucleus of the solitary tract to the lateral parabrachial nucleus) or any trigeminal stimulation in other locations (Chowdhury et al., 2014c). Also, interesting in this context is the development of the spinal cardiac reflex (Chowdhury and Schaller, 2017). Only recently, there could be found a more detail definition model of the TCR that included all these new findings (Meuwly et al., 2015b; Meuwly et al.).

In-addition, it was also investigated whether other skull base operations (excluding vestibular schwannomas) were accompanied by an intraoperative TCR occurrence: trans-sphenoidal surgery (Schaller, 2005a) and during Janetta operations (Schaller, 2005b) could be identified as further interventions aligned with a TCR. In these times, the issue of generalization of this already existing fragmented TCR knowledge has appeared in the (scientific) medical literature with regularity. Further research for TCR in different skull base approaches were forced to facilitate this generalization (Spiriev et al., 2011a,b) also pointing out the eminent importance of the TCR on the functional outcome after skull base surgery. Focusing on hearing and tinnitus function in patients with vestibular schwannoma has demonstrated the intraoperative hypotension owing to the TCR occurrence to be a negative prognostic factor for hearing preservation and postoperative tinnitus (Schaller et al., 2008b). Similarly, few reports also investigated various generally-based predisposing factors for TCR occurrences (Chowdhury et al., 2014a,b). These factors include hypercapnia, hypoxemia, light anesthesia, high resting vagal tone in children, narcotics such a sufentanil and alfentanil, preoperative β-blockers and calcium channel blockers (Meuwly et al., 2015a).

In a further stage of the TCR research, few surrogate models were developed to describe the better knowledge and understanding about the TCR behavior (Meuwly et al., 2015a,b, 2016; Meuwly et al.). These are not only useful to define and classify the TCR in a precise manner, but these also present the standardized definition of the TCR for the clinical research purposes. At this stage, the TCR phenomenon was also linked with various other problems including sudden infant death syndrome, sleep disorders (obstructive sleep apnea) and other neurological disorders (Chowdhury and Schaller; Golanov et al.; Singh et al.; Chowdhury and Schaller; Chowdhury et al.). This information opened the gate for further research of the TCR that was mainly highlighted during the intraoperative period.

Importantly, now it is known that the TCR physiology is not limited to surgical domain, its clinical implications are quite wide and variable. These manifestations can be from trivial to fatal as well as acute, sub-acute and even, chronic. In-addition, classical symptoms may not be present especially in chronic form of the TCR and make diagnosis even more challenging.

Therefore, the present research topic “The trigeminocardiac reflex: Beyond the diving reflex” imparts a new understanding of the TCR phenomenon and opens the gate for further research on this unique reflex for better understanding various neurological conditions and hopefully, would also assist in developing some treatment/interventions treat such conditions.

## Author contributions

All authors listed have made a substantial, direct and intellectual contribution to the work, and approved it for publication.

### Conflict of interest statement

The authors declare that the research was conducted in the absence of any commercial or financial relationships that could be construed as a potential conflict of interest.
